# Aqua­{2-(pyridin-2-yl)-*N*-[(pyridin-2-yl)methyl­idene]ethanamine-κ^3^ *N*,*N*′,*N*′′}(sulfato-κ^2^ *O*,*O*′)copper(II) tetra­hydrate

**DOI:** 10.1107/S1600536812049380

**Published:** 2012-12-08

**Authors:** Daniel Tinguiano, Mouhamadou Moustapha Sow, Farba Bouyagui Tamboura, Aliou Hamady Barry, Mohamed Gaye

**Affiliations:** a Département de Chimie, Faculté des Sciences et Techniques, Université Cheikh Anta Diop, Dakar, Senegal; b Département de Chimie, Faculté des Sciences, Université de Nouakchott, Nouakchott, Mauritania

## Abstract

The title complex, [Cu(SO_4_)(C_13_H_13_N_3_)(H_2_O)]·4H_2_O, was obtained by mixing copper sulfate penta­hydrate and 2-(pyridin-2-yl)-*N*-(pyridin-2-yl­methyl­idene)ethanamine in eth­anol under reflux conditions. The Cu^II^ ion shows a Jahn–Teller-distorted octa­hedral geometry, with equatorial positions occupied by three N atoms from the tridentate ligand (average Cu—N = 2.004 Å) and one O atom from a bidentate sulfate anion [Cu—O = 1.963 (2) Å]. The axial positions are occupied by one O atom from a coordinating water mol­ecule [Cu—O = 2.230 (3) Å] and one weakly bonded O atom [Cu—O = 2.750 (2) Å] from the bidentate sulfate ion. The complex mol­ecules are connected through O—H⋯O hydrogen bonds between the coordinating water mol­ecules and sulfate ions from neighboring complexes, forming a double chain parallel to the *c *axis. The chains are stabilized through additional hydrogen bonds by one of the non-coordinating water mol­ecules bridging between neighboring strands of the double chains. The remaining three water mol­ecules fill the inter­stitial space between the double chains and are involved in an intricate hydrogen-bonding network that consolidates the structure.

## Related literature

For related structures: see: de Bettencourt-Dias *et al.* (2010 ▶); Liu *et al.* (2010 ▶). For the Jahn–Teller effect, see: Jahn & Teller (1937 ▶).
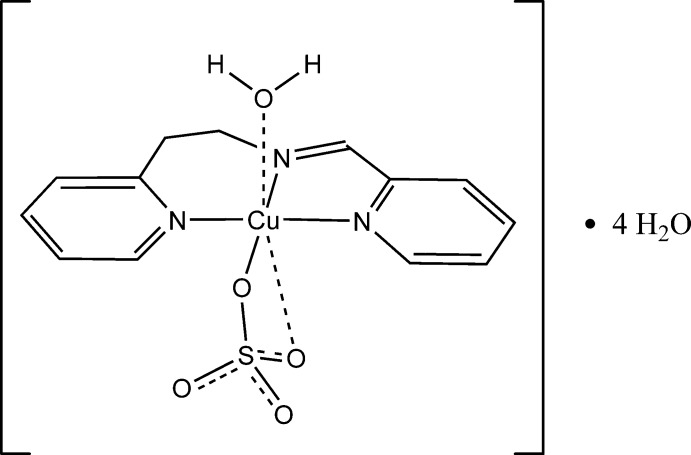


## Experimental

### 

#### Crystal data

[Cu(SO_4_)(C_13_H_13_N_3_)(H_2_O)]·4H_2_O
*M* *_r_* = 460.94
Monoclinic, 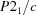

*a* = 10.7315 (17) Å
*b* = 23.605 (4) Å
*c* = 7.6478 (12) Å
β = 96.523 (3)°
*V* = 1924.8 (5) Å^3^
*Z* = 4
Mo *K*α radiation
μ = 1.29 mm^−1^
*T* = 293 K
0.10 × 0.07 × 0.05 mm

#### Data collection

Enraf–Nonius CAD-4 diffractometer
14560 measured reflections
3403 independent reflections
2620 reflections with *I* > 2σ(*I*)
*R*_int_ = 0.039
2 standard reflections every 60 min intensity decay: none

#### Refinement

*R*[*F* ^2^ > 2σ(*F* ^2^)] = 0.037
*wR*(*F* ^2^) = 0.103
*S* = 1.04
3403 reflections
274 parameters
17 restraints
H atoms treated by a mixture of independent and constrained refinement
Δρ_max_ = 0.57 e Å^−3^
Δρ_min_ = −0.39 e Å^−3^

### 

Data collection: *CAD-4 EXPRESS* (Enraf–Nonius, 1994 ▶); cell refinement: *CAD-4 EXPRESS*; data reduction: *MolEN* (Fair, 1990 ▶); program(s) used to solve structure: *SHELXS97* (Sheldrick, 2008 ▶); program(s) used to refine structure: *SHELXL97* (Sheldrick, 2008 ▶); molecular graphics: *ORTEP-3 for Windows* (Farrugia, 2012) ▶; software used to prepare material for publication: *SHELXL97*.

## Supplementary Material

Crystal structure: contains datablock(s) global. DOI: 10.1107/S1600536812049380/zl2518sup1.cif

Additional supplementary materials:  crystallographic information; 3D view; checkCIF report

## Figures and Tables

**Table 1:** Hydrogen-bond geometry (Å, °)

*D*—H⋯*A*	*D*—H	H⋯*A*	*D*⋯*A*	*D*—H⋯*A*
O5*W*—H5*WA*⋯O2^i^	0.80 (2)	1.97 (2)	2.765 (4)	172 (5)
O5*W*—H5*WB*⋯O1^ii^	0.79 (2)	2.04 (2)	2.803 (3)	162 (5)
O6*W*—H6*WA*⋯O8*W*	0.84 (2)	2.08 (2)	2.854 (9)	152 (5)
O6*W*—H6*WB*⋯O3	0.82 (2)	2.01 (2)	2.814 (5)	167 (8)
O7*W*—H7*WA*⋯O2^iii^	0.81 (2)	2.05 (2)	2.859 (5)	175 (6)
O7*W*—H7*WB*⋯O4	0.82 (2)	1.99 (2)	2.802 (5)	174 (7)
O8*W*—H8*WA*⋯O9*W*	0.83 (2)	2.11 (2)	2.890 (10)	156 (6)
O9*W*—H9*WA*⋯O7*W* ^iv^	0.84 (2)	1.91 (2)	2.705 (6)	158 (6)
O9*W*—H9*WB*⋯O6*W* ^i^	0.84 (2)	2.33 (2)	3.148 (8)	164 (7)

Symmetry codes: (i) 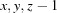; (ii) 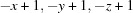; (iii) 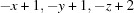; (iv) 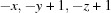.

**Fig. 1.**
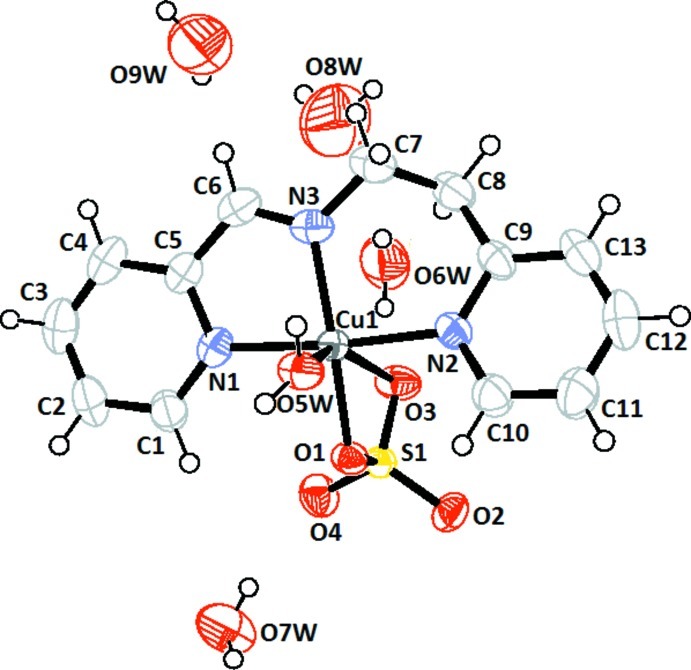
An ORTEP view of the title compound, showing the atom-numbering scheme. Displacement ellipsoids are plotted at the 50% probability level.

**Fig. 2.**
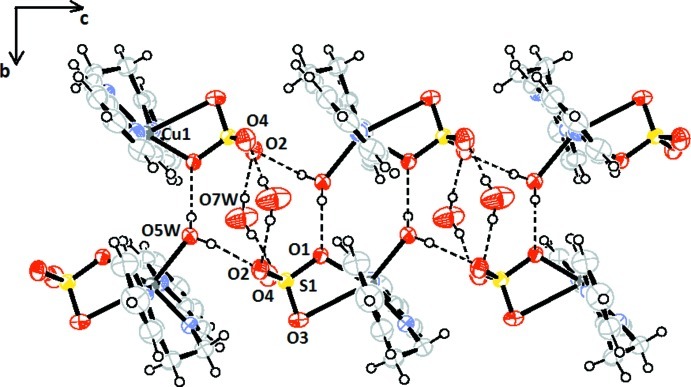
Molecular representation of the compound showing hydrogen bonds in the bc plane.

**Fig. 3.**
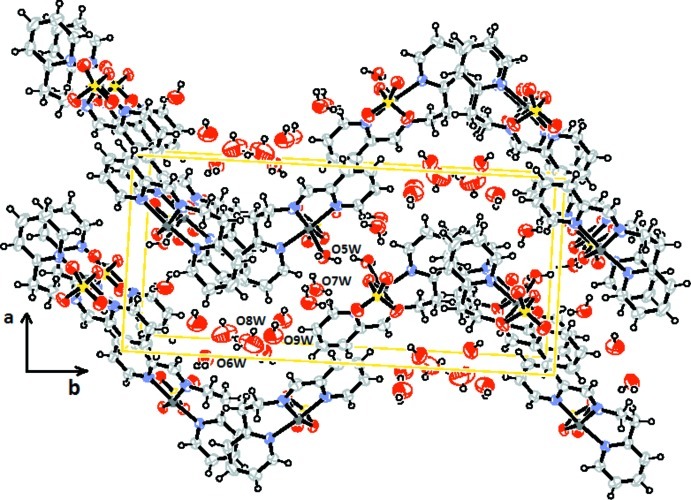
View of the structure along the c axis.

## supplementary crystallographic information

### Comment

The title complex, [Cu(SO_4_)(H_2_O)(C_13_H_13_N_3_)](H_2_O)_4_, was obtained by mixing copper sulfate pentahydrate and 2-(pyridin-2-yl)-*N*-(pyridin-2-ylmethylidene)ethanamine in ethanol under reflux conditions. It consists of a mononuclear Cu(II) complex and solvate water molecules with one neutral complex molecule and four not coordinated water molecules in the asymmetric unit (Fig. 1). The Cu(II) ion displays a six coordinated-geometry where the Cu atom is coordinated by three nitrogen atoms from the ligand molecule, two O atoms from the SO_4_^2-^ sulfate moiety and one O atom from a coordinated water molecule. The bond distances between the N atoms and the metal ion vary between 1.965 (3) Å [Cu1—N3] and 2.030 (3) Å [Cu1—N1]. These values are comparable to the bond lengths in a similar copper complex [1.971 (4)–2.021 (3) Å] (de Bettencourt-Dias *et al.*, 2010). The Cu—O bond distance for Cu1—O1 of the sulfate ion is 1.963 (2) Å, which is in the typical range (Liu *et al.*, 2010). The remaining positions are occupied by one O atom from a coordinated water molecule (Cu—O = 2.230 (3) Å) and one weakly coordinated O (Cu—O = 2.750 (2) Å) atom from the bidentate sulfate ion. The angle between the central metal ion and the O atoms [O1—Cu1—O5W] is equal to 98.24 (10) °. The angles between the Cu^II^ ion and the coordinating N atoms vary between 80.77 (13) ° [N3—Cu1—N1] and 174.29 (12) ° [N2—Cu1—N1]. The Cu(II) center of the molecule complex thus adopts a distorted octahedral geometry. Atoms [N1, N2, N3, O1] are arranged in the equatorial plane with some deviations from the ideal geometry. The axial positions are occupied by the oxygen atom of the coordinated water molecule and one O atom from the bidentate sulfate. The axial bond lengths between the Cu^II^ ion and the O atoms are considerably longer than the equatorial bond distances between the Cu^II^ ion and the O atom of the sulfate ligand as a consequence of the Jahn–Teller effect (Jahn & Teller, 1937).

The sulfate anion has a slightly distorted tetrahedral geometry due to the fact that two of the oxygen atoms of the sulfate group are coordinated to the metal center, with one of the Cu—O distances being considerably longer than the other one (1.963 (2) and 2.750 (2) Å). The S—O bond lengths (S—O4 = 1.450 (3); S—O3 = 1.459 (3); S—O2 = 1.462 (3) and S—O1 = 1.517 (2) Å) indicate a S—O single bond for the tightly copper bonded O atom and S—O bonds between single and double bond character for the other three. The O—S—O angles, which range from 107.01 (15) to 111.23 (16) °, are close to the ideal tetrahedral angle value of 109.5 °.

Neighboring [Cu(SO_4_)(H_2_O)(C_13_H_13_N_3_)] units are connected with each other through hydrogen bonds creating double chains that stretch parallel to the *c* axis direction (Fig 2, Fig. 3). The coordinated water molecules are connected with complexes through OW—H···O—SO_3_ hydrogen bonds between O5W and O2 and O1 of neighboring complexes' sulfate ions. The double chains thus created are in addition connected with each other through O—H···O hydrogen bonds mediated by the uncoordinated water molecule of O7W which acts as bridge between two sulfate groups of two molecules belonging to parallel strands of the double chains (SO_3_—O···H—OW—H···O—SO_3_) (Table 1). The interstitial space between the double chains is filled by the three remaining lattice water molecules which are involved in an intricate hydrogen bonding network that consolidates the crystal packing.

### Experimental

2-(Pyridin-2-yl)-*N*-(pyridin-2-ylmethylidene)ethanamine (0.2111 g, 1 mmol) was dissolved in 10 ml of ethanol. To the resulting solution, Cu(SO_4_).5H_2_O (0.2497 g, 1 mmol) was added. The mixture was stirred at room temperature for 2 h. The green solution was filtered off and left for slow evaporation. Crystals that separated from the green solution, were filtered off and recrystallized from dimethylformamide. On standing for two weeks, crystals suitable for X-ray diffraction analysis were obtained. Yield: 74.5%. Anal. Calc. for [C_13_H_23_N_3_O_9_SCu] (%): C, 33.87; H, 5.03; N, 9.12; S, 6.96. Found: C, 33.84; H, 5.01; N, 9.09; S, 6.98. Decomposition point: 305 °C. Selected IR data (cm^-1^, KBr pellet): 3445 s (ν_OH_), 1646 s (ν_C__═__N_), 1598 m, 1109 m (ν_as_SO_4_), 774 m.

### Refinement

H atoms of the water molecule were located in the Fourier difference maps and refined with O—H distance restraints of 0.82 (2) Å. Additional H···O distance restraints were used for H atoms of two water molecules (O8W and O9W). H atoms of =CH and CH_2_ groups were placed geometrically and refined using a riding model with C—H distantces between 0.93 and 0.97 Å with *U*_iso_(H) = 1.2*U*_eq_(C).

### Crystal data

**Table:**

[Cu(SO_4_)(C_13_H_13_N_3_)(H_2_O)]·4H_2_O	*F*(000) = 956
*M**_r_* = 460.94	*D*_x_ = 1.591 Mg m^−^^3^
Monoclinic, *P*2_1_/*c*	Mo *K*α radiation, λ = 0.71073 Å
Hall symbol: -P 2ybc	Cell parameters from 25 reflections
*a* = 10.7315 (17) Å	θ = 11–15°
*b* = 23.605 (4) Å	µ = 1.29 mm^−^^1^
*c* = 7.6478 (12) Å	*T* = 293 K
β = 96.523 (3)°	Prismatic, blue
*V* = 1924.8 (5) Å^3^	0.10 × 0.07 × 0.05 mm
*Z* = 4	

### Data collection

**Table:**

Enraf–Nonius CAD-4 diffractometer	*R*_int_ = 0.039
Radiation source: fine-focus sealed tube	θ_max_ = 25.1°, θ_min_ = 1.7°
Graphite monochromator	*h* = −12→12
π scans	*k* = −28→28
14560 measured reflections	*l* = −8→9
3403 independent reflections	2 standard reflections every 60 min
2620 reflections with *I* > 2σ(*I*)	intensity decay: none

### Refinement

**Table:**

Refinement on *F*^2^	Primary atom site location: structure-invariant direct methods
Least-squares matrix: full	Secondary atom site location: difference Fourier map
*R*[*F*^2^ > 2σ(*F*^2^)] = 0.037	Hydrogen site location: inferred from neighbouring sites
*wR*(*F*^2^) = 0.103	H atoms treated by a mixture of independent and constrained refinement
*S* = 1.04	*w* = 1/[σ^2^(*F*_o_^2^) + (0.0448*P*)^2^ + 2.5709*P*] where *P* = (*F*_o_^2^ + 2*F*_c_^2^)/3
3403 reflections	(Δ/σ)_max_ = 0.001
274 parameters	Δρ_max_ = 0.57 e Å^−^^3^
17 restraints	Δρ_min_ = −0.39 e Å^−^^3^

### Special details

**Table:**

Geometry. All e.s.d.'s (except the e.s.d. in the dihedral angle between two l.s. planes) are estimated using the full covariance matrix. The cell e.s.d.'s are taken into account individually in the estimation of e.s.d.'s in distances, angles and torsion angles; correlations between e.s.d.'s in cell parameters are only used when they are defined by crystal symmetry. An approximate (isotropic) treatment of cell e.s.d.'s is used for estimating e.s.d.'s involving l.s. planes.
Refinement. Refinement of *F*^2^ against ALL reflections. The weighted *R*-factor *wR* and goodness of fit *S* are based on *F*^2^, conventional *R*-factors *R* are based on *F*, with *F* set to zero for negative *F*^2^. The threshold expression of *F*^2^ > σ(*F*^2^) is used only for calculating *R*-factors(gt) *etc*. and is not relevant to the choice of reflections for refinement. *R*-factors based on *F*^2^ are statistically about twice as large as those based on *F*, and *R*- factors based on ALL data will be even larger.

### Fractional atomic coordinates and isotropic or equivalent isotropic displacement parameters (Å^2^)

**Table:**

	*x*	*y*	*z*	*U*_iso_*/*U*_eq_	
Cu1	0.34621 (4)	0.409141 (17)	0.46361 (5)	0.03121 (15)	
S1	0.34297 (8)	0.41815 (3)	0.84451 (11)	0.0315 (2)	
N1	0.2175 (3)	0.47177 (13)	0.4132 (4)	0.0385 (7)	
N2	0.4622 (3)	0.34185 (12)	0.4987 (4)	0.0362 (7)	
N3	0.2371 (3)	0.37299 (13)	0.2717 (4)	0.0405 (7)	
O1	0.4070 (2)	0.44192 (9)	0.6934 (3)	0.0348 (6)	
O2	0.4420 (2)	0.39896 (11)	0.9786 (3)	0.0437 (6)	
O3	0.2646 (3)	0.37097 (11)	0.7753 (4)	0.0485 (7)	
O4	0.2680 (3)	0.46243 (11)	0.9121 (4)	0.0506 (7)	
O5W	0.4827 (3)	0.45044 (11)	0.3049 (3)	0.0424 (6)	
H5WA	0.473 (4)	0.4385 (19)	0.207 (3)	0.064*	
H5WB	0.498 (4)	0.4833 (9)	0.301 (6)	0.064*	
O6W	0.0350 (4)	0.3126 (2)	0.7184 (8)	0.1132 (17)	
H6WA	0.038 (5)	0.308 (4)	0.609 (3)	0.170*	
H6WB	0.103 (4)	0.328 (3)	0.719 (10)	0.170*	
O7W	0.3003 (3)	0.57344 (17)	1.0390 (7)	0.0916 (13)	
H7WA	0.372 (3)	0.583 (2)	1.034 (10)	0.137*	
H7WB	0.287 (6)	0.5420 (15)	0.996 (9)	0.137*	
O8W	−0.0444 (6)	0.2896 (3)	0.3565 (9)	0.158 (2)	
H8WA	−0.086 (7)	0.307 (5)	0.275 (8)	0.237*	
H8WB	−0.026 (12)	0.2552 (16)	0.348 (9)	0.237*	
O9W	−0.1188 (4)	0.3475 (2)	0.0284 (9)	0.1241 (18)	
H9WA	−0.185 (5)	0.367 (3)	0.025 (11)	0.186*	
H9WB	−0.086 (7)	0.332 (3)	−0.054 (9)	0.186*	
C1	0.2128 (4)	0.52248 (16)	0.4895 (6)	0.0470 (10)	
H1	0.2746	0.5324	0.5795	0.056*	
C2	0.1173 (4)	0.56075 (18)	0.4370 (6)	0.0567 (12)	
H2	0.1172	0.5963	0.4893	0.068*	
C3	0.0239 (4)	0.5461 (2)	0.3089 (6)	0.0580 (12)	
H3	−0.0415	0.5711	0.2754	0.070*	
C4	0.0278 (4)	0.4941 (2)	0.2304 (6)	0.0541 (11)	
H4	−0.0351	0.4831	0.1433	0.065*	
C5	0.1269 (4)	0.45822 (17)	0.2829 (5)	0.0430 (9)	
C6	0.1431 (4)	0.40272 (18)	0.2065 (5)	0.0482 (10)	
H6	0.0874	0.3893	0.1138	0.058*	
C7	0.2575 (4)	0.31678 (16)	0.2077 (5)	0.0503 (10)	
H7A	0.3207	0.3179	0.1266	0.060*	
H7B	0.1804	0.3024	0.1449	0.060*	
C8	0.3000 (4)	0.27820 (16)	0.3600 (5)	0.0500 (10)	
H8A	0.2429	0.2824	0.4487	0.060*	
H8B	0.2942	0.2393	0.3189	0.060*	
C9	0.4305 (4)	0.28867 (14)	0.4440 (5)	0.0392 (9)	
C10	0.5785 (4)	0.35136 (17)	0.5752 (5)	0.0471 (10)	
H10	0.5999	0.3879	0.6124	0.056*	
C11	0.6683 (4)	0.3095 (2)	0.6015 (7)	0.0625 (12)	
H11	0.7486	0.3177	0.6544	0.075*	
C12	0.6360 (5)	0.2554 (2)	0.5476 (7)	0.0686 (14)	
H12	0.6942	0.2262	0.5645	0.082*	
C13	0.5164 (5)	0.24484 (17)	0.4684 (6)	0.0547 (11)	
H13	0.4936	0.2084	0.4314	0.066*	

### Atomic displacement parameters (Å^2^)

**Table:**

	*U*^11^	*U*^22^	*U*^33^	*U*^12^	*U*^13^	*U*^23^
Cu1	0.0333 (3)	0.0289 (2)	0.0306 (2)	−0.00165 (18)	−0.00006 (17)	−0.00066 (17)
S1	0.0346 (5)	0.0302 (4)	0.0292 (4)	−0.0022 (4)	0.0015 (4)	−0.0004 (3)
N1	0.0333 (17)	0.0444 (18)	0.0375 (17)	0.0019 (14)	0.0036 (14)	0.0055 (14)
N2	0.0410 (18)	0.0299 (16)	0.0383 (17)	−0.0020 (13)	0.0066 (14)	0.0004 (13)
N3	0.0452 (19)	0.0429 (18)	0.0338 (17)	−0.0106 (15)	0.0058 (15)	−0.0040 (14)
O1	0.0449 (15)	0.0314 (13)	0.0277 (13)	−0.0065 (11)	0.0029 (11)	−0.0003 (10)
O2	0.0460 (16)	0.0490 (16)	0.0342 (14)	0.0025 (12)	−0.0033 (12)	0.0082 (11)
O3	0.0494 (16)	0.0459 (16)	0.0499 (16)	−0.0195 (13)	0.0044 (13)	−0.0039 (12)
O4	0.0543 (17)	0.0475 (16)	0.0516 (17)	0.0106 (13)	0.0135 (14)	−0.0051 (13)
O5W	0.0552 (17)	0.0403 (15)	0.0321 (14)	−0.0134 (13)	0.0071 (13)	0.0004 (12)
O6W	0.073 (3)	0.067 (3)	0.190 (5)	−0.012 (2)	−0.029 (3)	0.005 (3)
O7W	0.053 (2)	0.075 (3)	0.150 (4)	−0.0019 (18)	0.021 (3)	−0.035 (3)
O8W	0.113 (4)	0.190 (7)	0.169 (6)	0.042 (5)	0.009 (4)	−0.019 (5)
O9W	0.068 (3)	0.108 (4)	0.190 (6)	0.001 (3)	−0.012 (3)	−0.029 (4)
C1	0.045 (2)	0.039 (2)	0.056 (3)	0.0024 (18)	0.004 (2)	0.0026 (18)
C2	0.054 (3)	0.038 (2)	0.079 (3)	0.011 (2)	0.014 (3)	0.009 (2)
C3	0.042 (3)	0.059 (3)	0.073 (3)	0.012 (2)	0.012 (2)	0.025 (2)
C4	0.040 (2)	0.071 (3)	0.050 (3)	0.008 (2)	−0.0033 (19)	0.016 (2)
C5	0.039 (2)	0.049 (2)	0.040 (2)	0.0007 (18)	0.0015 (17)	0.0068 (18)
C6	0.046 (2)	0.059 (3)	0.037 (2)	−0.004 (2)	−0.0036 (18)	0.0018 (19)
C7	0.062 (3)	0.044 (2)	0.045 (2)	−0.012 (2)	0.009 (2)	−0.0068 (18)
C8	0.062 (3)	0.036 (2)	0.052 (2)	−0.0040 (19)	0.008 (2)	−0.0066 (18)
C9	0.052 (2)	0.0287 (18)	0.038 (2)	−0.0014 (17)	0.0127 (18)	0.0010 (15)
C10	0.045 (2)	0.040 (2)	0.056 (3)	−0.0015 (18)	0.006 (2)	0.0012 (18)
C11	0.046 (3)	0.061 (3)	0.079 (3)	0.010 (2)	−0.001 (2)	0.002 (2)
C12	0.067 (3)	0.054 (3)	0.086 (4)	0.027 (2)	0.013 (3)	0.008 (3)
C13	0.074 (3)	0.032 (2)	0.060 (3)	0.008 (2)	0.016 (2)	−0.0025 (19)

### Geometric parameters (Å, º)

**Table:**

Cu1—O1	1.963 (2)	C1—C2	1.390 (5)
Cu1—N3	1.965 (3)	C1—H1	0.9300
Cu1—N2	2.017 (3)	C2—C3	1.364 (6)
Cu1—N1	2.030 (3)	C2—H2	0.9300
Cu1—O5W	2.230 (3)	C3—C4	1.370 (6)
S1—O4	1.450 (3)	C3—H3	0.9300
S1—O3	1.459 (3)	C4—C5	1.383 (5)
S1—O2	1.462 (3)	C4—H4	0.9300
S1—O1	1.517 (2)	C5—C6	1.453 (6)
N1—C1	1.335 (5)	C6—H6	0.9300
N1—C5	1.349 (5)	C7—C8	1.508 (6)
N2—C10	1.336 (5)	C7—H7A	0.9700
N2—C9	1.354 (4)	C7—H7B	0.9700
N3—C6	1.282 (5)	C8—C9	1.494 (6)
N3—C7	1.440 (5)	C8—H8A	0.9700
O5W—H5WA	0.796 (19)	C8—H8B	0.9700
O5W—H5WB	0.793 (19)	C9—C13	1.384 (5)
O6W—H6WA	0.844 (19)	C10—C11	1.379 (6)
O6W—H6WB	0.822 (17)	C10—H10	0.9300
O7W—H7WA	0.81 (2)	C11—C12	1.374 (7)
O7W—H7WB	0.817 (19)	C11—H11	0.9300
O8W—H8WA	0.828 (19)	C12—C13	1.378 (7)
O8W—H8WB	0.84 (2)	C12—H12	0.9300
O9W—H9WA	0.840 (19)	C13—H13	0.9300
O9W—H9WB	0.843 (18)		
			
O1—Cu1—N3	161.62 (12)	C2—C3—C4	119.0 (4)
O1—Cu1—N2	93.10 (11)	C2—C3—H3	120.5
N3—Cu1—N2	93.65 (13)	C4—C3—H3	120.5
O1—Cu1—N1	91.87 (11)	C3—C4—C5	118.9 (4)
N3—Cu1—N1	80.77 (13)	C3—C4—H4	120.6
N2—Cu1—N1	174.28 (12)	C5—C4—H4	120.6
O1—Cu1—O5W	98.24 (10)	N1—C5—C4	122.3 (4)
N3—Cu1—O5W	98.95 (11)	N1—C5—C6	113.7 (3)
N2—Cu1—O5W	89.05 (11)	C4—C5—C6	124.0 (4)
N1—Cu1—O5W	93.05 (11)	N3—C6—C5	117.5 (4)
O4—S1—O3	111.05 (17)	N3—C6—H6	121.2
O4—S1—O2	111.23 (16)	C5—C6—H6	121.2
O3—S1—O2	111.19 (16)	N3—C7—C8	109.8 (3)
O4—S1—O1	108.81 (15)	N3—C7—H7A	109.7
O3—S1—O1	107.35 (14)	C8—C7—H7A	109.7
O2—S1—O1	107.01 (15)	N3—C7—H7B	109.7
C1—N1—C5	118.4 (3)	C8—C7—H7B	109.7
C1—N1—Cu1	129.0 (3)	H7A—C7—H7B	108.2
C5—N1—Cu1	112.7 (2)	C9—C8—C7	114.8 (3)
C10—N2—C9	118.7 (3)	C9—C8—H8A	108.6
C10—N2—Cu1	117.2 (2)	C7—C8—H8A	108.6
C9—N2—Cu1	124.0 (3)	C9—C8—H8B	108.6
C6—N3—C7	121.0 (3)	C7—C8—H8B	108.6
C6—N3—Cu1	115.4 (3)	H8A—C8—H8B	107.5
C7—N3—Cu1	123.6 (3)	N2—C9—C13	120.8 (4)
S1—O1—Cu1	113.77 (13)	N2—C9—C8	118.4 (3)
Cu1—O5W—H5WA	110 (3)	C13—C9—C8	120.8 (4)
Cu1—O5W—H5WB	127 (3)	N2—C10—C11	123.1 (4)
H5WA—O5W—H5WB	109 (5)	N2—C10—H10	118.4
H6WA—O6W—H6WB	85 (6)	C11—C10—H10	118.4
H7WA—O7W—H7WB	112 (3)	C12—C11—C10	118.2 (4)
H8WA—O8W—H8WB	122 (10)	C12—C11—H11	120.9
H9WA—O9W—H9WB	130 (9)	C10—C11—H11	120.9
N1—C1—C2	121.3 (4)	C11—C12—C13	119.4 (4)
N1—C1—H1	119.3	C11—C12—H12	120.3
C2—C1—H1	119.3	C13—C12—H12	120.3
C3—C2—C1	120.0 (4)	C12—C13—C9	119.7 (4)
C3—C2—H2	120.0	C12—C13—H13	120.1
C1—C2—H2	120.0	C9—C13—H13	120.1

### Hydrogen-bond geometry (Å, º)

**Table:**

*D*—H···*A*	*D*—H	H···*A*	*D*···*A*	*D*—H···*A*
O5*W*—H5*WA*···O2^i^	0.80 (2)	1.97 (2)	2.765 (4)	172 (5)
O5*W*—H5*WB*···O1^ii^	0.79 (2)	2.04 (2)	2.803 (3)	162 (5)
O6*W*—H6*WA*···O8*W*	0.84 (2)	2.08 (2)	2.854 (9)	152 (5)
O6*W*—H6*WB*···O3	0.82 (2)	2.01 (2)	2.814 (5)	167 (8)
O7*W*—H7*WA*···O2^iii^	0.81 (2)	2.05 (2)	2.859 (5)	175 (6)
O7*W*—H7*WB*···O4	0.82 (2)	1.99 (2)	2.802 (5)	174 (7)
O8*W*—H8*WA*···O9*W*	0.83 (2)	2.11 (2)	2.890 (10)	156 (6)
O9*W*—H9*WA*···O7*W*^iv^	0.84 (2)	1.91 (2)	2.705 (6)	158 (6)
O9*W*—H9*WB*···O6*W*^i^	0.84 (2)	2.33 (2)	3.148 (8)	164 (7)
C1—H1···O1	0.93	2.66	3.107 (5)	111
C6—H6···O9*W*	0.93	2.44	3.254 (6)	146
C7—H7*A*···O2^i^	0.97	2.64	3.398 (5)	135
C10—H10···O1	0.93	2.57	3.025 (5)	111

Symmetry codes: (i) *x*, *y*, *z*−1; (ii) −*x*+1, −*y*+1, −*z*+1; (iii) −*x*+1, −*y*+1, −*z*+2; (iv) −*x*, −*y*+1, −*z*+1.
